# Supplementary material for: Cold-shock proteome of myoblasts reveals role of RBM3 in promotion of mitochondrial metabolism and myoblast differentiation
Source: Commun Biol. 2024 Apr 30;7:515. doi: 10.1038/s42003-024-06196-4 (PMC11061143; doi:10.1038/s42003-024-06196-4)
Supplement: Supplementary file 2 — supplementary material [file 42003_2024_6196_MOESM2_ESM.pdf]

Figure S1

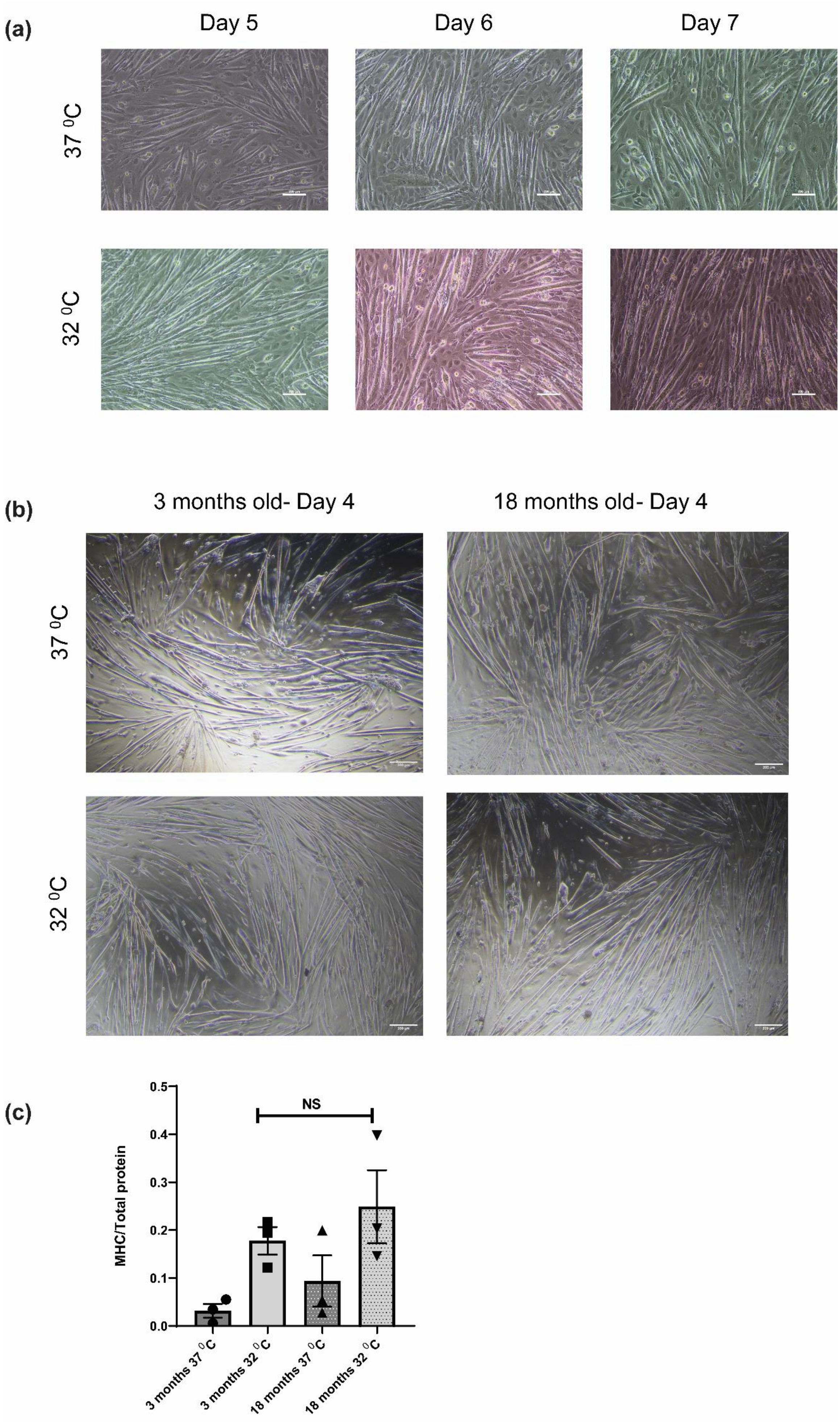
(a)
Day 5
Day 6
Day 7
37 ⁰C
32 ⁰C
(b)
3 months old- Day 4
18 months old- Day 4
37 ⁰C
32 ⁰C
(c)
MHC/Total protein
0.5
0.4
0.3
0.2
0.1
0.0
NS
3 months 37 ⁰C
3 months 32 ⁰C
18 months 37 ⁰C
18 months 32 ⁰C

Figure S1. (a) Representative brightfield images of C2C12 myoblasts differentiated for 5, 6 and 7 days at 37 ^{0}C after hypothermic pre-conditioning at 32 ^{0}C for 72 hours, (10X, Scale bar:100um). **(b)** Representative brightfield images of primary myoblasts (from 3 and 18-month-old mice) differentiated for 4 days at 37 ^{0}C after hypothermic pre-conditioning at 32 ^{0}C for 72 hours (4X, Scale bar:100um). **(c)** Bar graph quantifying the protein levels of MHC using primary myoblasts (from 3 and 18-month-old mice) differentiated for 4 days at 37 ^{0}C after hypothermic pre-conditioning. Y-axis represents the intensity of MHC normalized to total protein (n=3). Error bar in the bar graph represents SEM. Significance tested by two-tailed student's t-test (unpaired) where NS represents non-significant.

Figure S2

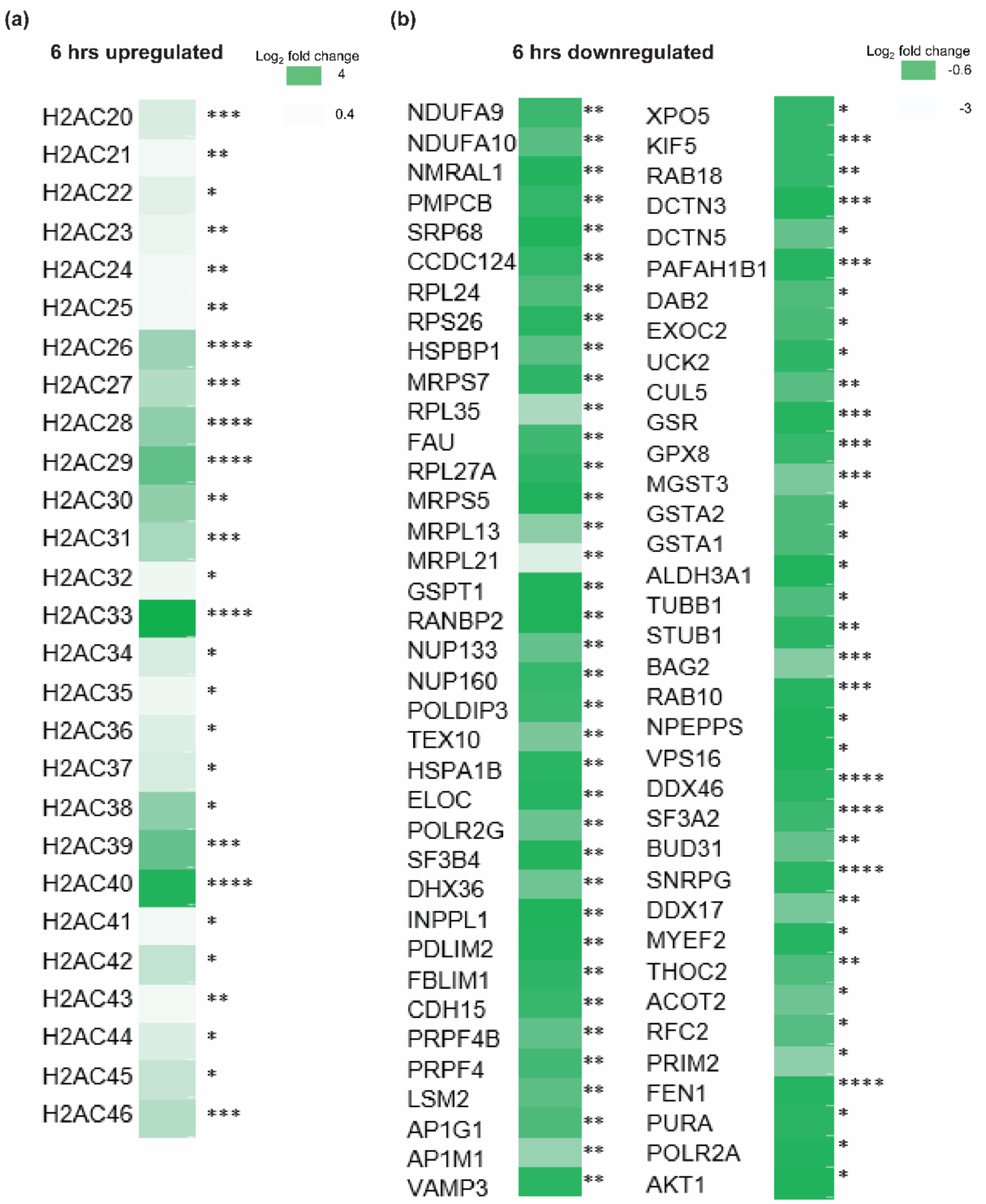

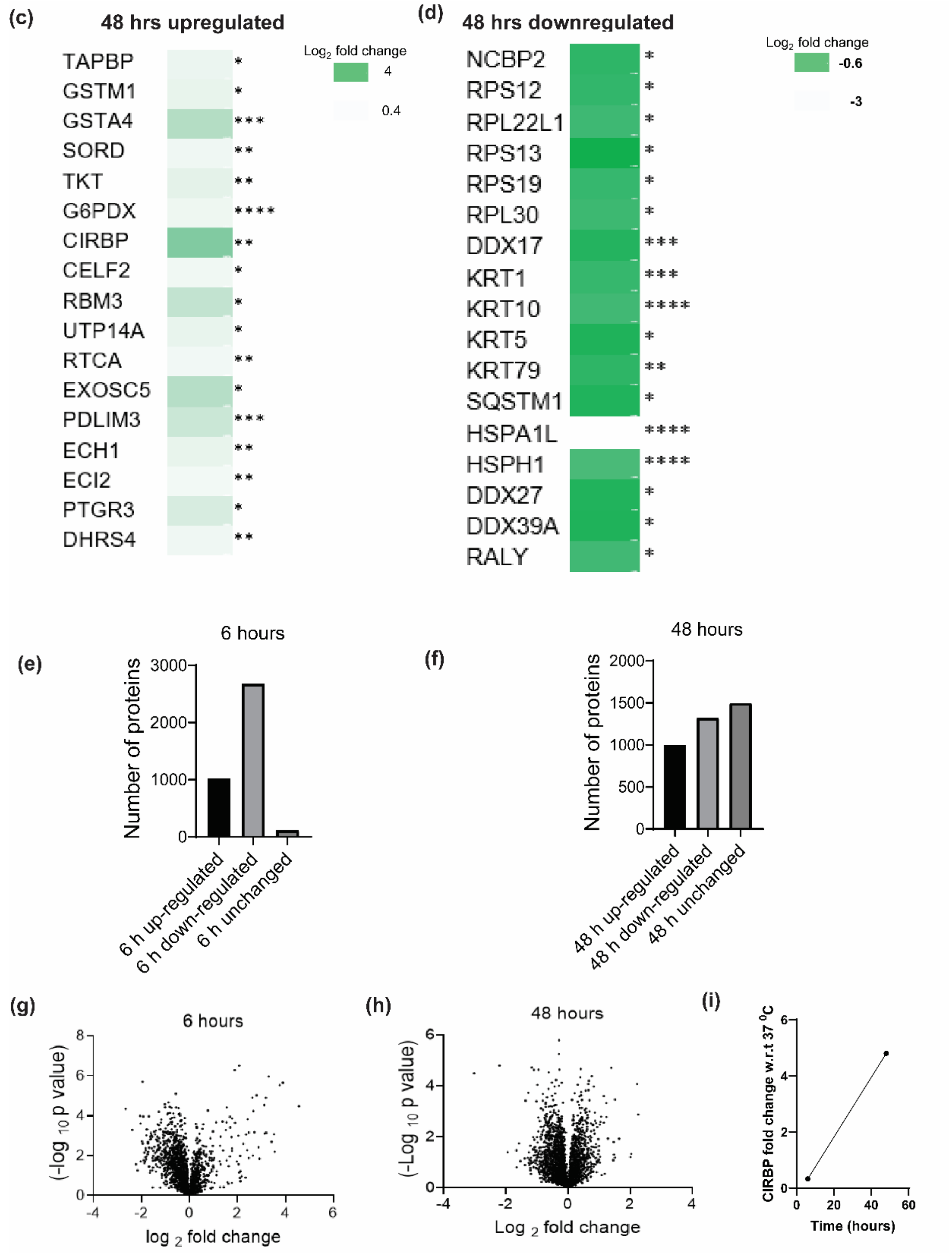

Figure S2. (a) Heat map of upregulated proteins (fold change >= 1.5) in C2C12 myoblasts at 32 ^{0}C for 6 hours (n=3). **(b)** Heat map of downregulated proteins (fold change <= 0.6) in C2C12 myoblasts at 32 ^{0}C for 6 hours (n=3). **(c)** Heat map of upregulated proteins (fold change >= 1.5) in C2C12 myoblasts at 32 ^{0}C for 48 hours (n=3). **(d)** Heat map of downregulated proteins (fold change <= 0.6) in C2C12 at 32 ^{0}C for 48 hours (n=3). **(e)** Bar graph showing the number of upregulated, downregulated and unchanged proteins in C2C12 myoblasts at 32 ^{0}C for 6 hours (n=2). **(f)** Bar graph showing the number of upregulated, downregulated and unchanged proteins in C2C12 myoblasts at 32 ^{0}C for 48 hours. Volcano plot of proteomics data from C2C12 myoblasts exposed to 32 ^{0}C for 6 hours **(g)** and 48 hours **(h)** depicting log $_2$ fold change vs – log $_{10}$ p value. **(i)** Line graph representing the protein level of CIRBP at different time

points (6 hours and 48 hours), where x-axis represents time in hours and y-axis represents the fold change in the protein levels of CIRBP at 32 ^{0}C with respect to 37 ^{0}C *, **, *** represents p value < 0.05, 0.01 and 0.001 respectively.

Figure S3

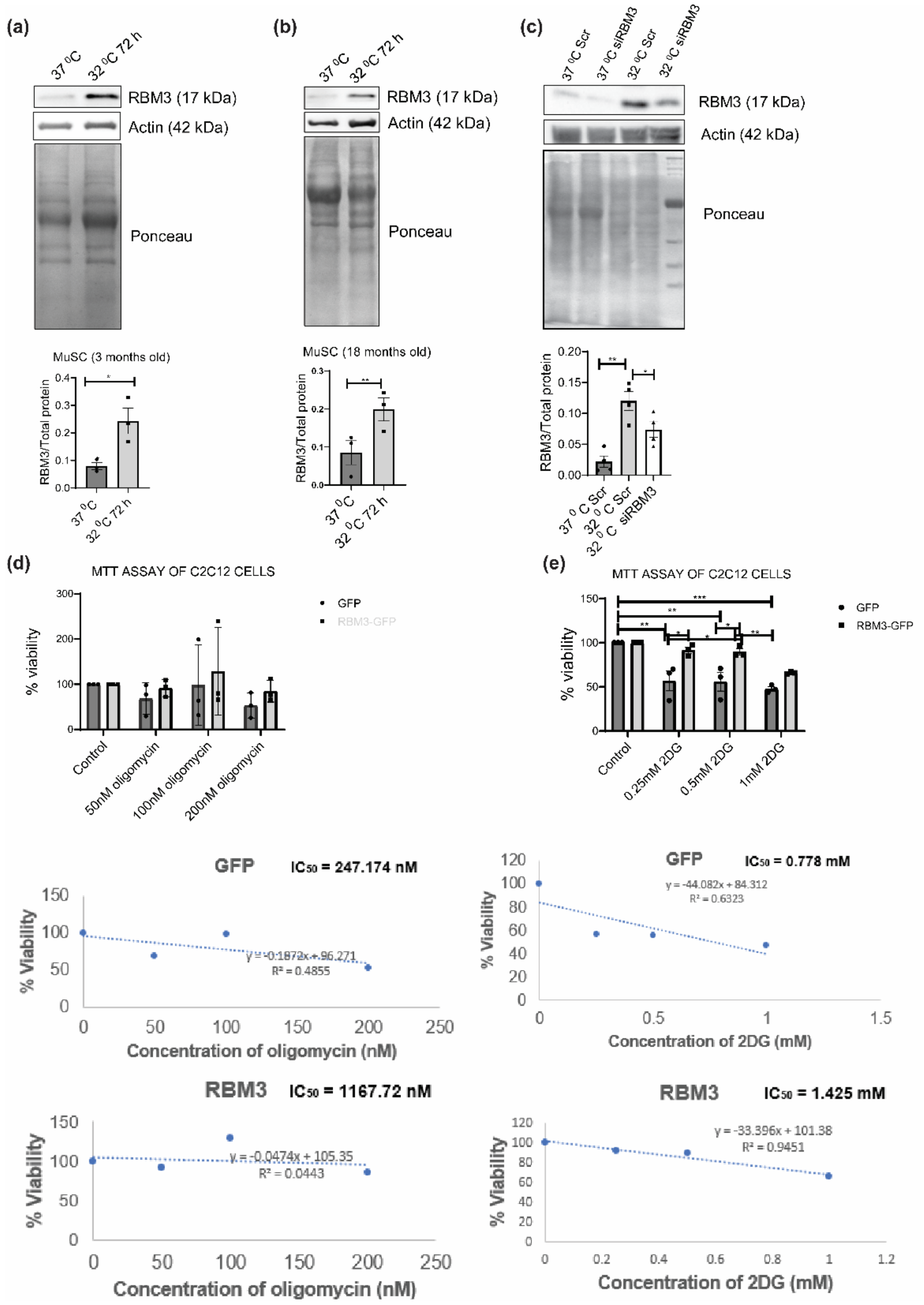

Figure S3. (a) Western blot of protein levels of RBM3 in primary myoblasts (from 3-month-old mice) incubated at 37 ^{0}C and 32 ^{0}C (72 hours) respectively. Bar graph representing the protein levels of RBM3 quantified from the western blot normalized to total protein levels (n=3). **(b)** Western blot of protein levels of RBM3 in primary myoblasts (from 18-month-old mice) incubated at 37 ^{0}C and 32 ^{0}C (72 hours) respectively. Bar graph representing the protein levels

of RBM3 (quantified from the western blot) normalized to total protein levels (n=3). Significance calculated using two-tailed student's t-test (paired). **(c)** Western blot of protein levels of RBM3 in C2C12 transfected with Scr or siRBM3 at 37 ^{0}C and 32 ^{0}C (72 hrs) respectively. Bar graph representing the protein levels of RBM3 quantified from the western blot normalized to the total protein levels at 37 ^{0}C Scr and 32 ^{0}C (72 hours), respectively (n=4). **(d)** Bar graph showing the % viability of C2C12 cells overexpressing RBM3-GFP and GFP respectively, in the presence of different concentrations of oligomycin (50nM, 100nM and 200nM). IC_{50} plot for oligomycin using C2C12 cells overexpressing RBM3-GFP and GFP respectively, where the x-axis represents the oligomycin concentration and the y-axis represents the %viability of the cells. (n=3) Significance tested by two-way ANOVA. **(e)** Bar graph showing the % viability of C2C12 cells overexpressing RBM3-GFP and GFP respectively, in presence of different concentrations of 2DG (0.25mM, 0.5mM and 1mM). IC_{50} plot for 2DG using C2C12 cells overexpressing RBM3-GFP and GFP respectively, where the x-axis represents the oligomycin concentration and the y-axis represents the %viability of the cells. (n=3). Error bar in the bar graph represents SEM. *, **, *** represents p value < 0.05, 0.01 and 0.001 respectively.

Figure S4

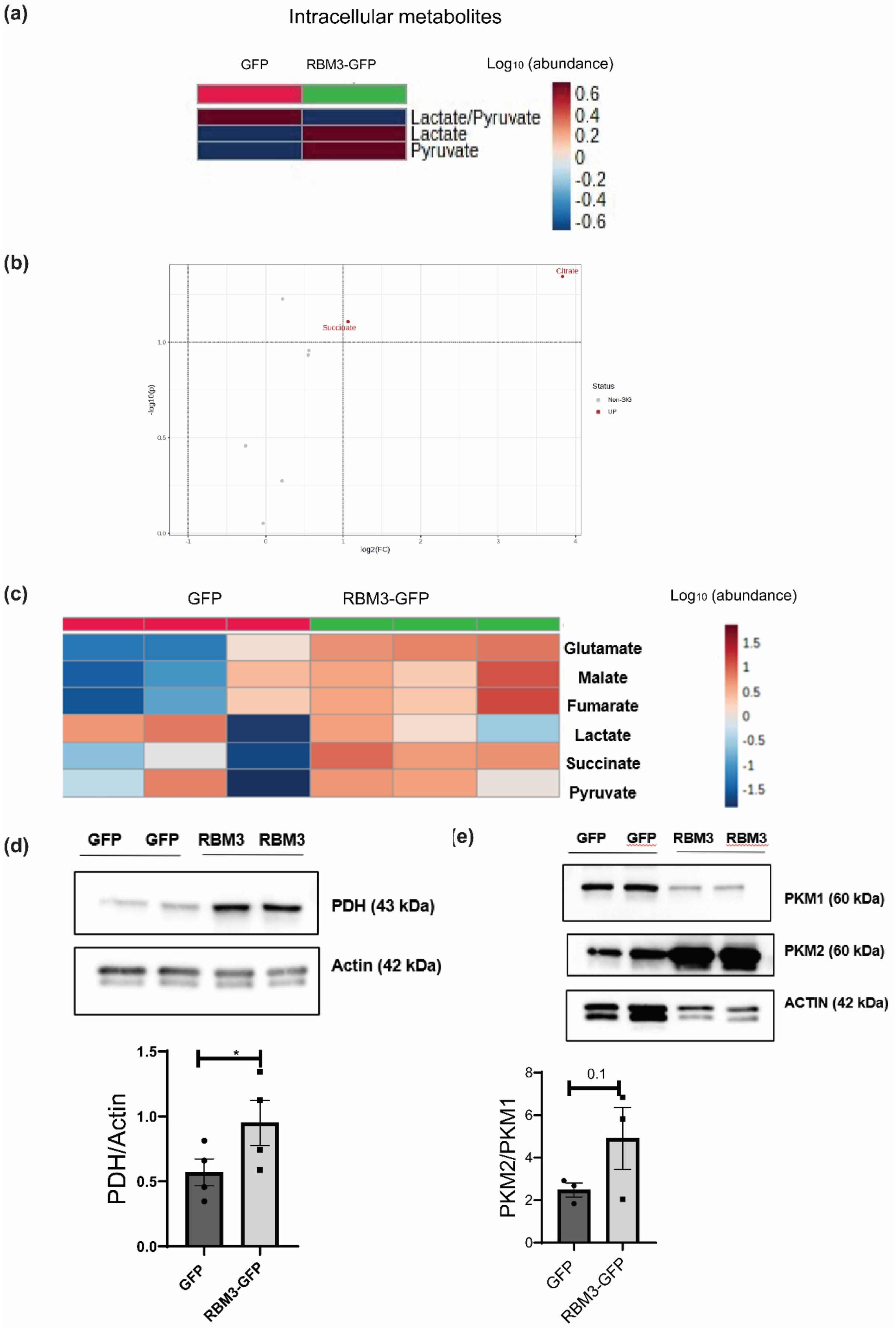

Figure S4. (a) Heat map showing the ratio of the intracellular levels of lactate to pyruvate

using C2C12 cells overexpressing RBM3-GFP and GFP respectively (n=3). **(b)** Volcano plot of Intracellular metabolites of C2C12 cells overexpressing RBM3-GFP and GFP respectively, where the x-axis represents the log of fold change and the y-axis represents -log of p value. Grey dots represent non-significant metabolite species and blue dot represents significantly high levels of metabolite species (n=3). **(c)** Heat map showing intracellular levels of TCA metabolites using C2C12 cells overexpressing RBM3-GFP and GFP respectively (n=3). **(d)** Western blot of the protein levels of PDH using C2C12 cells overexpressing RBM3-GFP and GFP, (n=3). Bar graph quantifying the protein levels of PDH normalized to that of actin. **(e)** Western blot analysis of levels of PKM1 and PKM2 using C2C12 cells overexpressing RBM3-GFP and GFP respectively (n=3). Error bar in the bar graph represents SEM. Significance tested by two-tailed student's t-test (paired). *, **, *** represents p value < 0.05, 0.01 and 0.001 respectively.

Figure S5

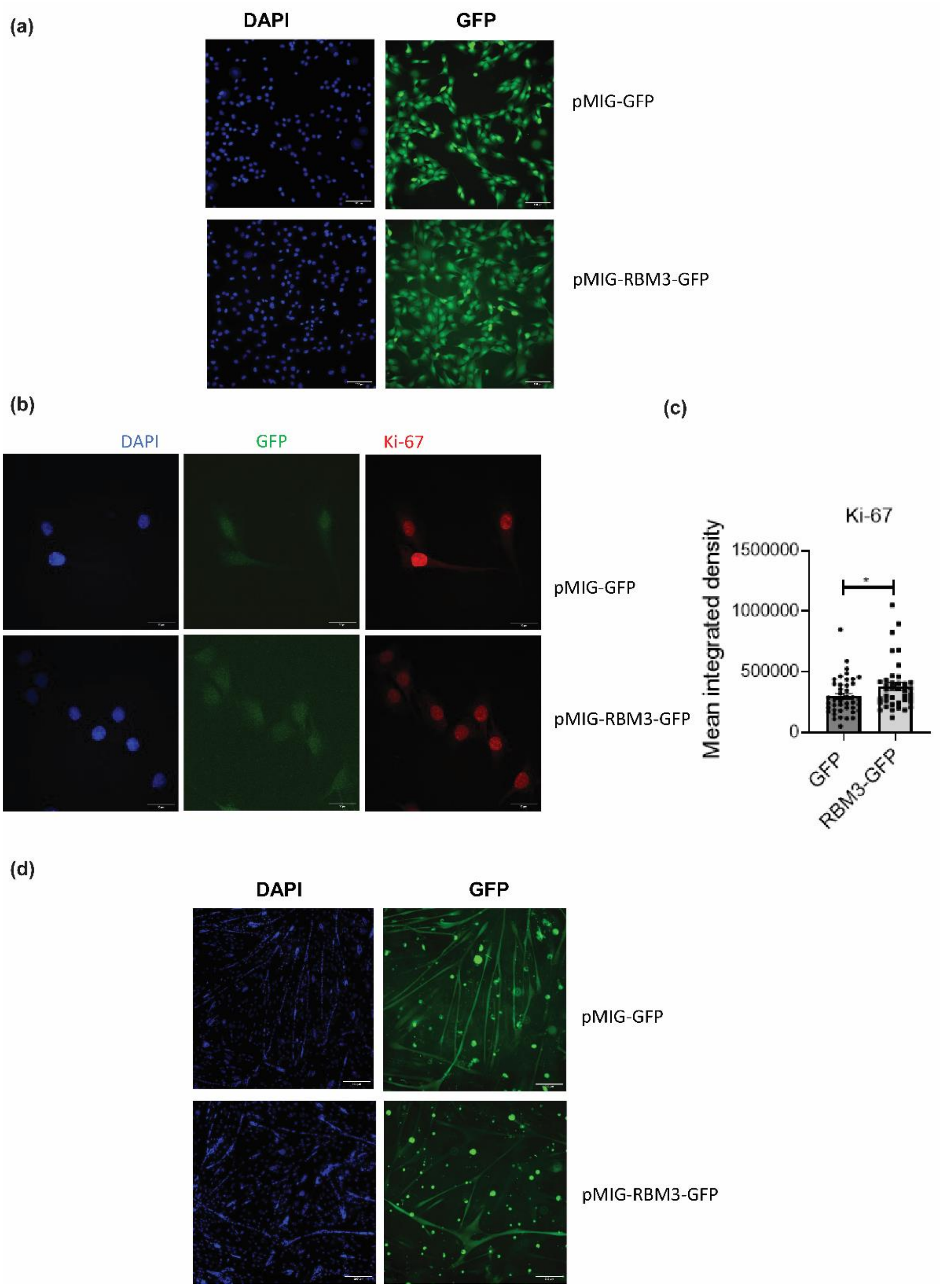

Figure S5. (a) Representative epifluorescence images of stable C2C12 cells transduced with pMIG-RBM3-GFP and pMIG-GFP. **(b)** C2C12 cells overexpressing RBM3-GFP and GFP respectively at 37 ^{0}C, immunostained with Ki-67 antibody. Red indicates Ki-67 staining, blue indicates DAPI and Green indicate GFP (Scale bar: 30um). **(c)** Bar graph representing the quantitation of Ki-67 staining where the y-axis represents the mean integrated density of Ki-67 positive nucleus stain quantified from b (41 individual data points from 2 individual experiments). **(d)** Representative epifluorescence images of primary myoblasts from 3-month-old mice transfected with pMIG-RBM3 and pMIG-GFP, differentiated for 4 days. Error bar in the bar graph represents SEM. Significance tested by two-tailed student's t-test (unpaired). *, **, *** represents p value < 0.05, 0.01 and 0.001 respectively.

Figure S6

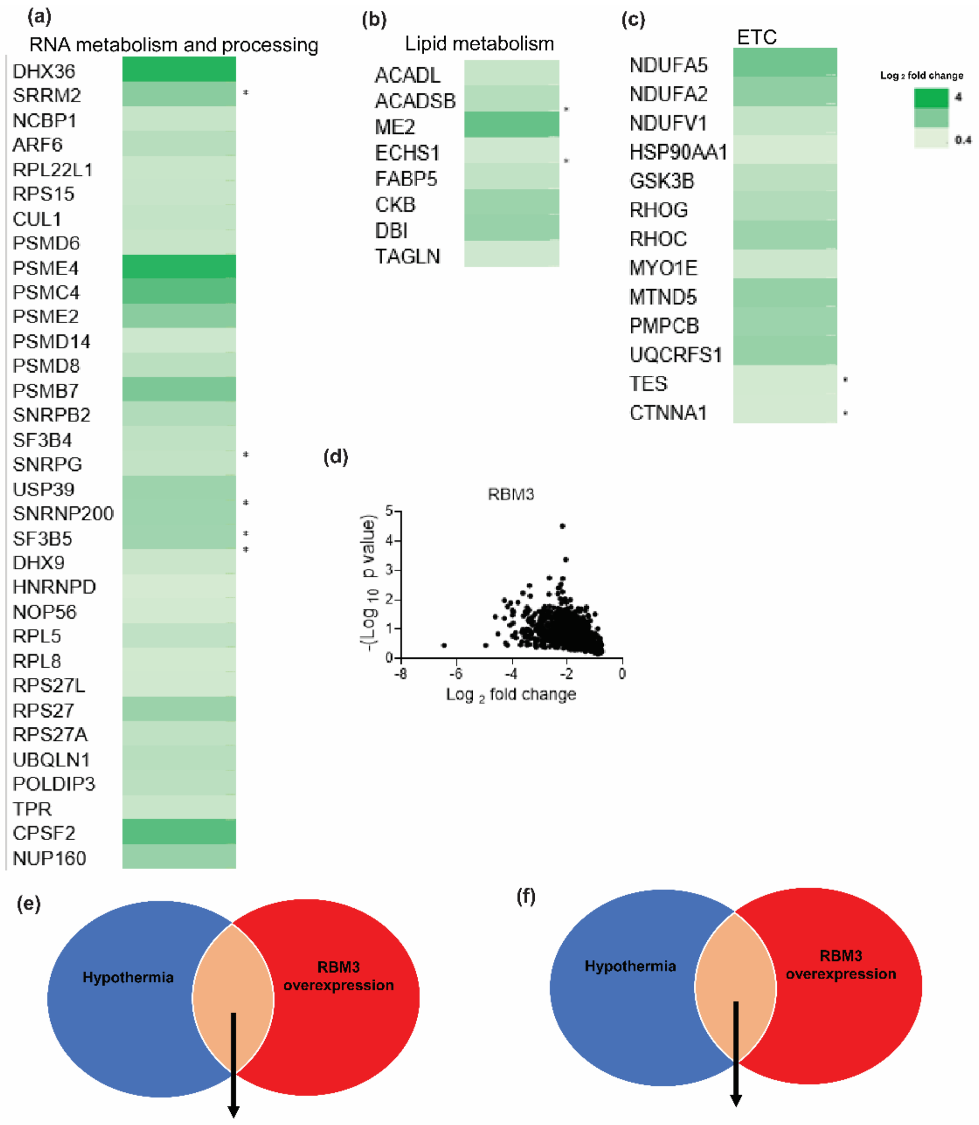

- Monocarboxylic acid metabolic processes
- Adaptive immune response
- Melanin biosynthetic process
- Fatty acid metabolism
- Platelet aggregation
- mRNA processing

- Metabolism of RNA
- Amyotrophic lateral sclerosis
- Golgi-Endoplasmic reticulum transport
- Intra-cellular protein transport
- Establishment of protein localization to membrane
- Nuclear-cytoplasmic transport

Figure S6. (a) Heat map of upregulated proteins (fold change >= 1.5) involved in RNA processing and metabolism in C2C12 cells overexpressing RBM3 (n=3). **(b)** Heat map of upregulated proteins (fold change >= 1.5) involved in lipid metabolism in C2C12 cells overexpressing RBM3 (n=3). **(c)** Heat map of upregulated proteins (fold change >= 1.5) involved in ETC in C2C12 cells overexpressing RBM3 (n=3). **(d)** Volcano plot of proteomics data from C2C12 myoblasts overexpressing RBM3-GFP depicting log $_2$fold change vs – log $_{10}$ p value. **(e)** Venn diagram showing the GO pathway enrichment analysis of overlapping pathways in hypothermia and C2C12 cells overexpressing RBM3 using set of proteins which are increased in abundance. **(f)** Venn diagram showing the GO pathway enrichment analysis of overlapping pathways hypothermia and C2C12 cells overexpressing RBM3 using set of proteins which are decreased in abundance. (n=3). Significance tested by two-tailed student's t-test (unpaired). *, **, *** represents p value < 0.05, 0.01 and 0.001 respectively.

Figure S7

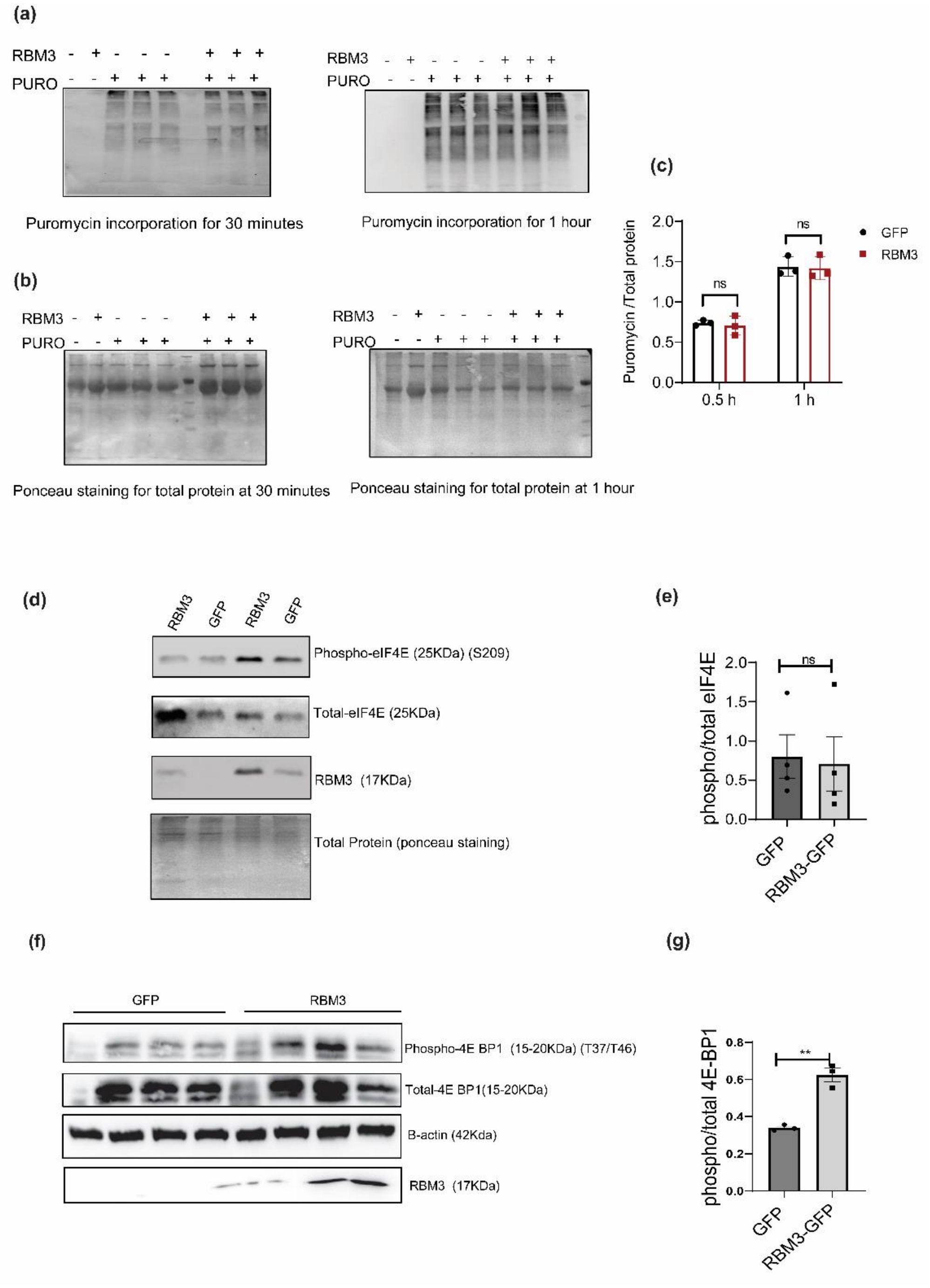

Figure S7. (a) Western blots analysis showing overall protein synthesis in C2C12 cells overexpressing RBM3-GFP and GFP respectively, after (i) 30 minutes and (ii) 1 hour of puromycin (PURO) incorporation into proteins (n=3)**. (b)** Ponceau staining of the western blots analysis in A, after (i) 30 minutes and (ii) 1 hour of puromycin (PURO) incorporation into proteins. **(c)** Bar graph quantifying the levels of puromycin incorporation normalized to total protein (n=3)**.** Error bar in the bar graph represents SD. Significance tested by two-way ANOVA. **(d)** Western blot analysis of eIF4E using C2C12 cells overexpressing RBM3-GFP and GFP respectively (n=4). **(e)** Bar graph quantifying the ratio of phosphorylated to total eIF4E in C2C12 cells (n=4). **(f)** Western blot analysis of 4E-BP1 using C2C12 cells overexpressing RBM3-GFP and GFP respectively (n=3). **(g)** Bar graph quantifying the ratio of phosphorylated to total 4E-BP1 in C2C12 cells (n=3)**.** Error bar in the bar graph represents SEM. Significance tested by two-tailed student's t-test (unpaired). *, **, *** represents p value < 0.05, 0.01 and 0.001 respectively and NS represents non-significant.

Figure S8

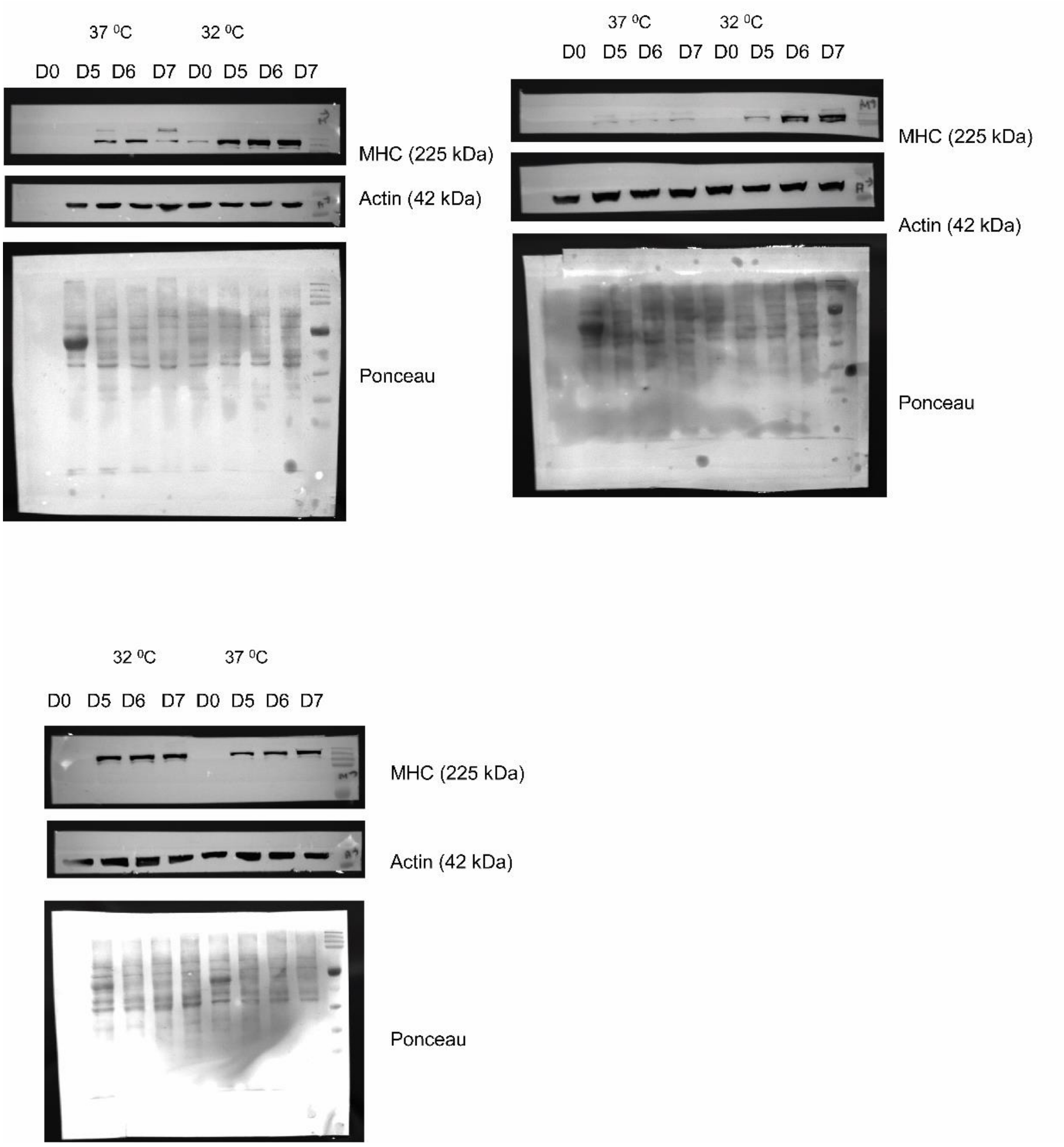

Figure: Uncropped raw western blot images of Fig 1a, 1b

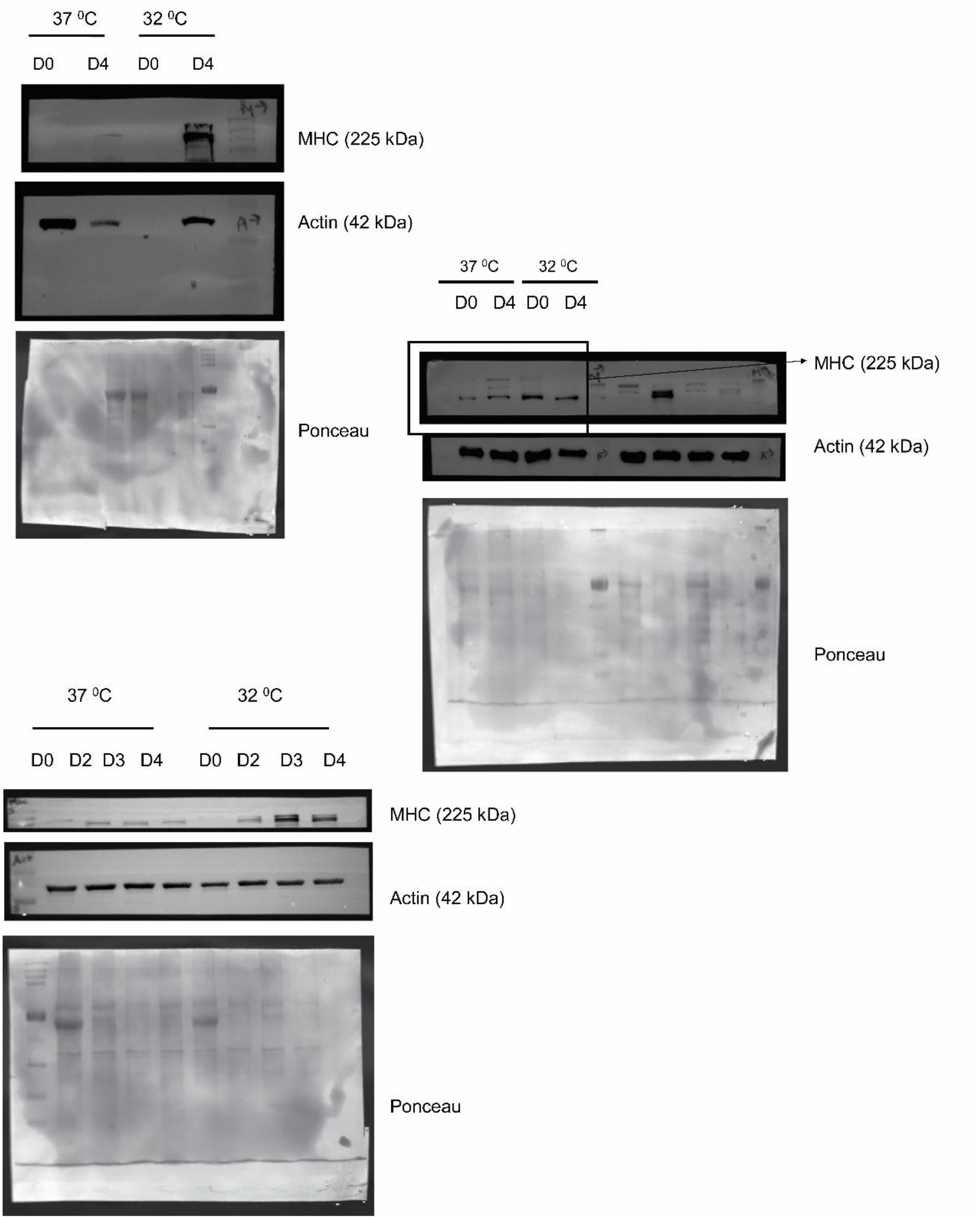

Figure: Uncropped raw western blot images of Fig 1g, 1h

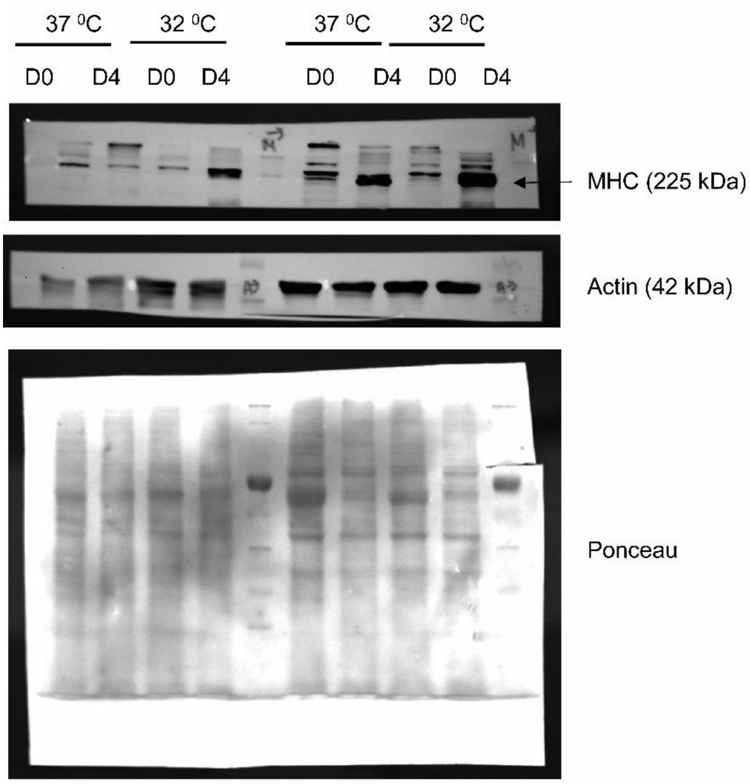

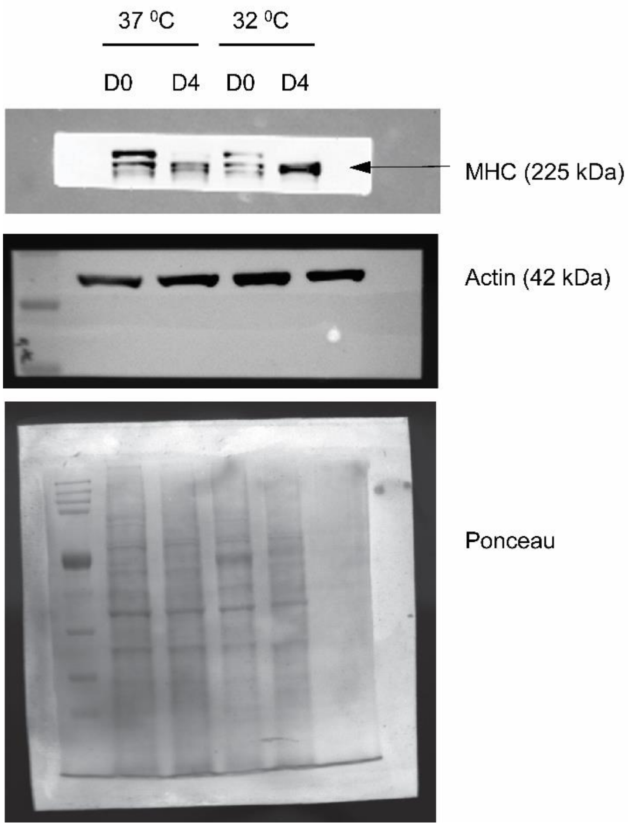

Figure: Uncropped raw western blot images of Fig 1i, 1j

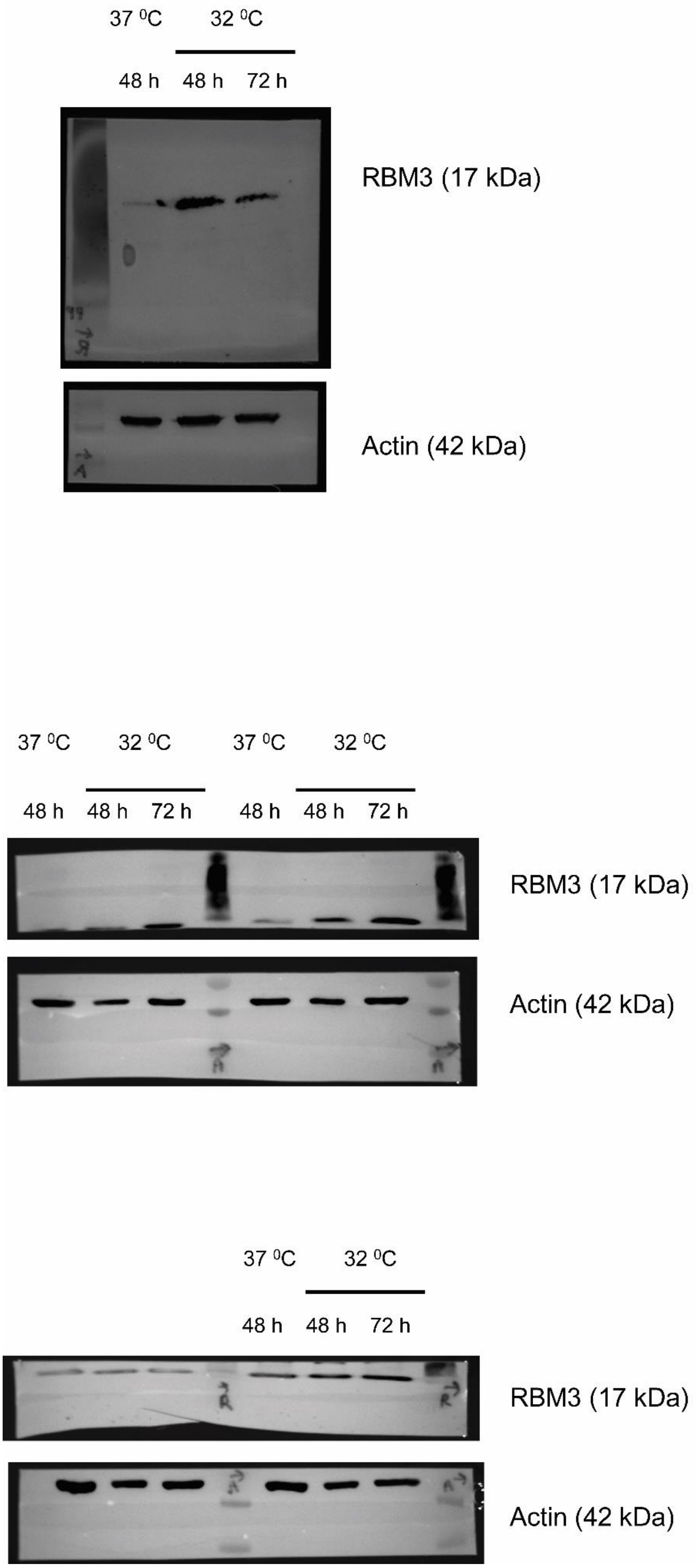

Figure: Uncropped raw western blot images of Fig 4b, 4c

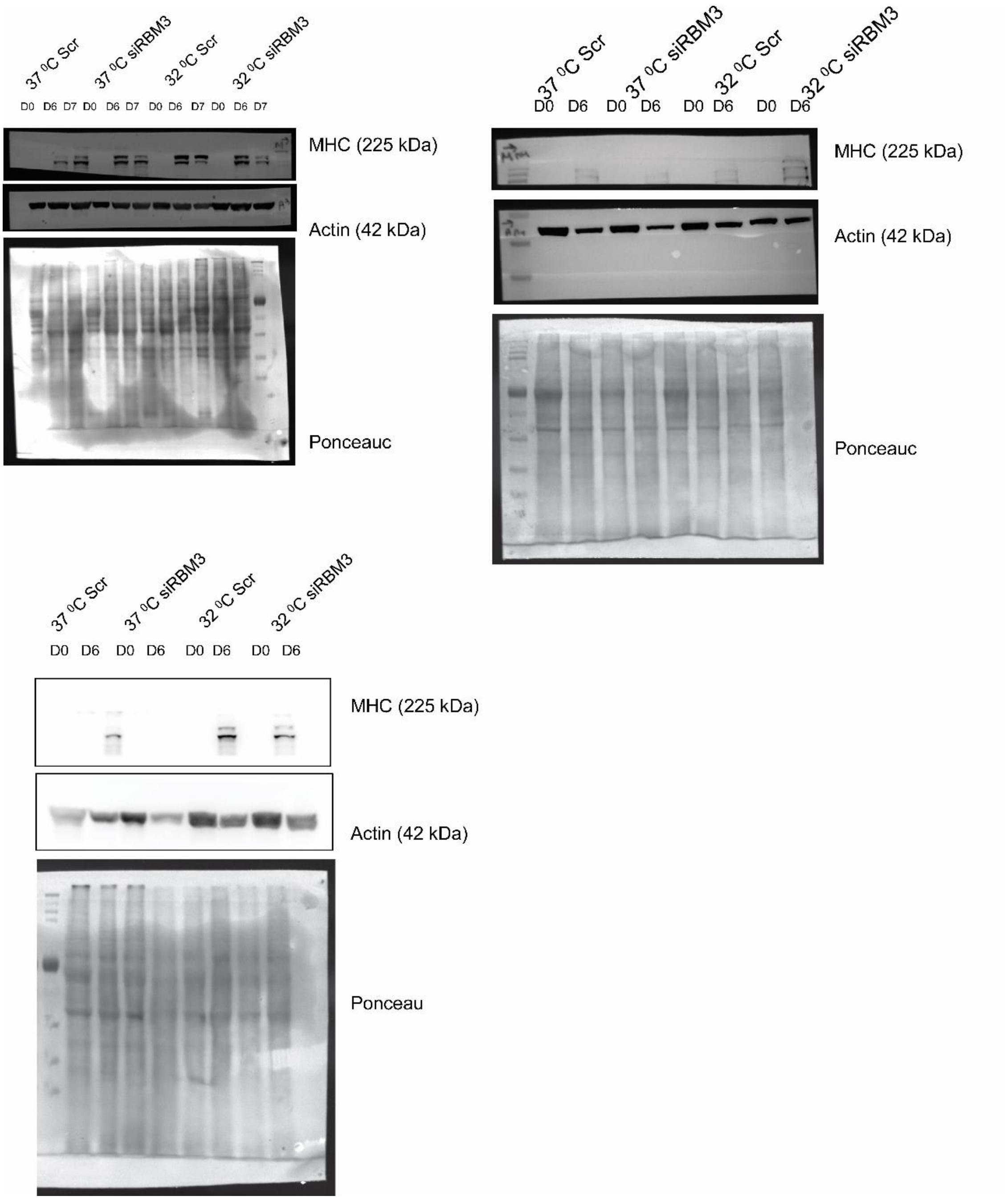

Figure: Uncropped raw western blot images of Fig 4d, 4e

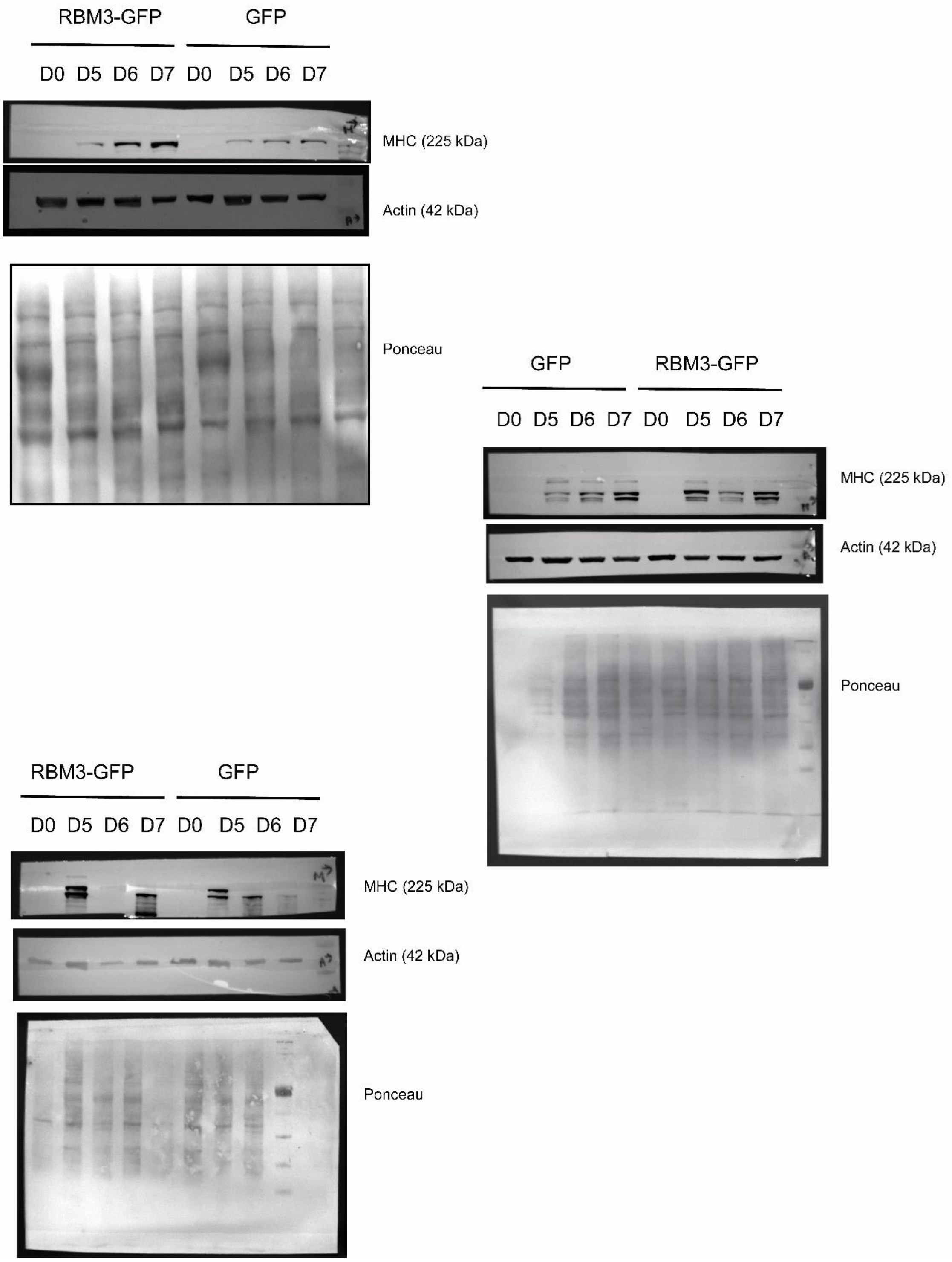

Figure: Uncropped raw western blot images of Fig 5i, 5j

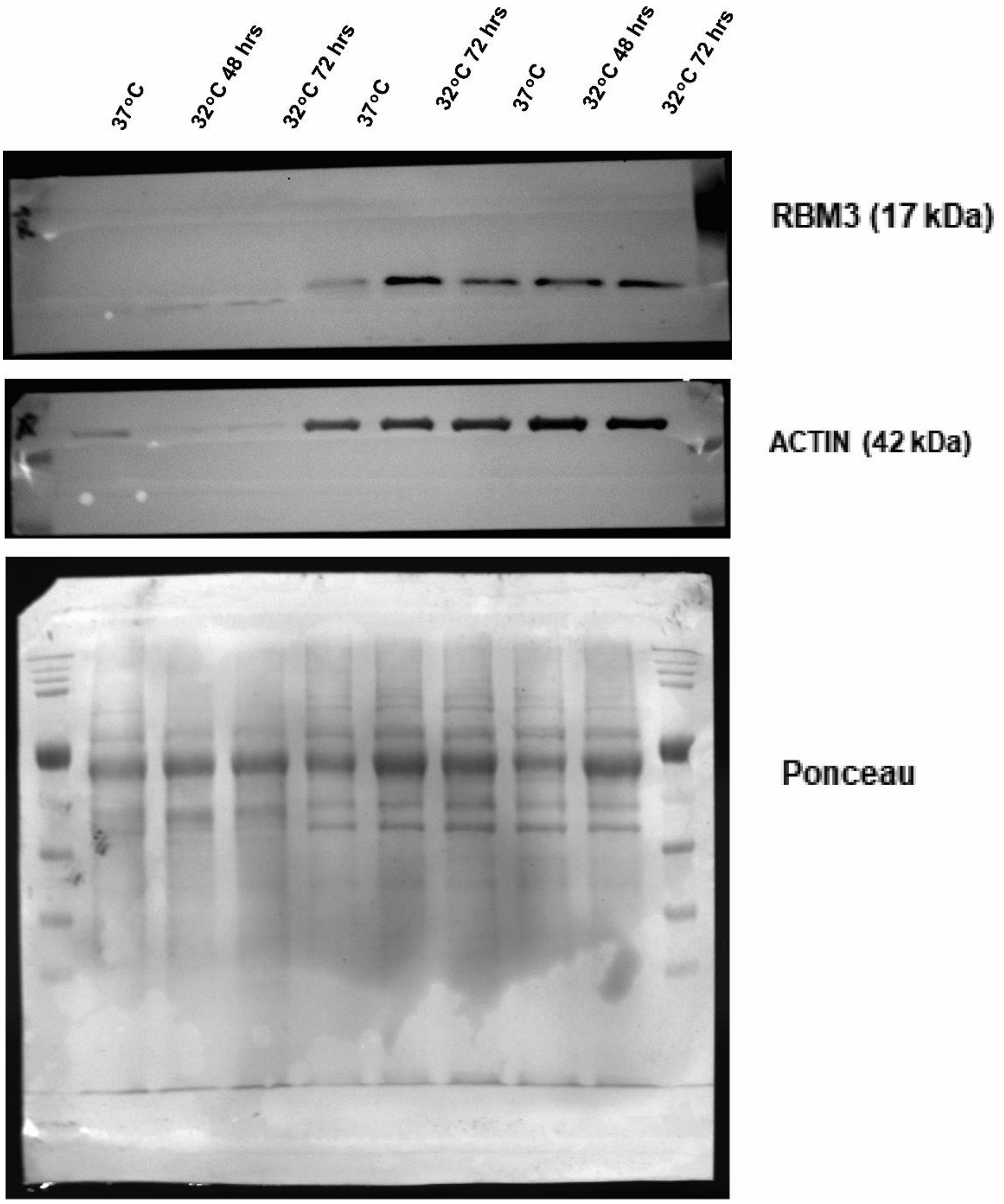

Figure: Uncropped raw western blot images of Fig S3a

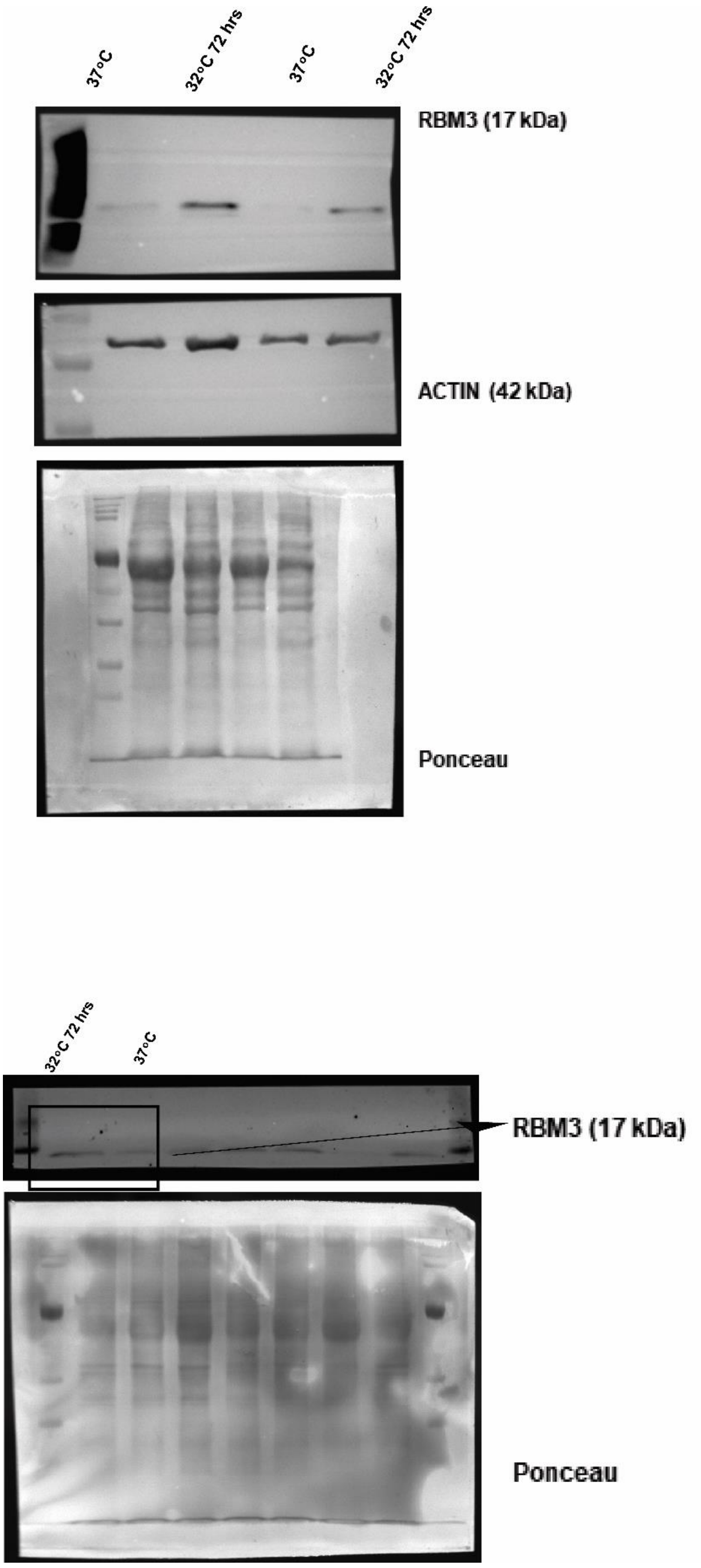

Figure: Uncropped raw western blot images of Fig S3b

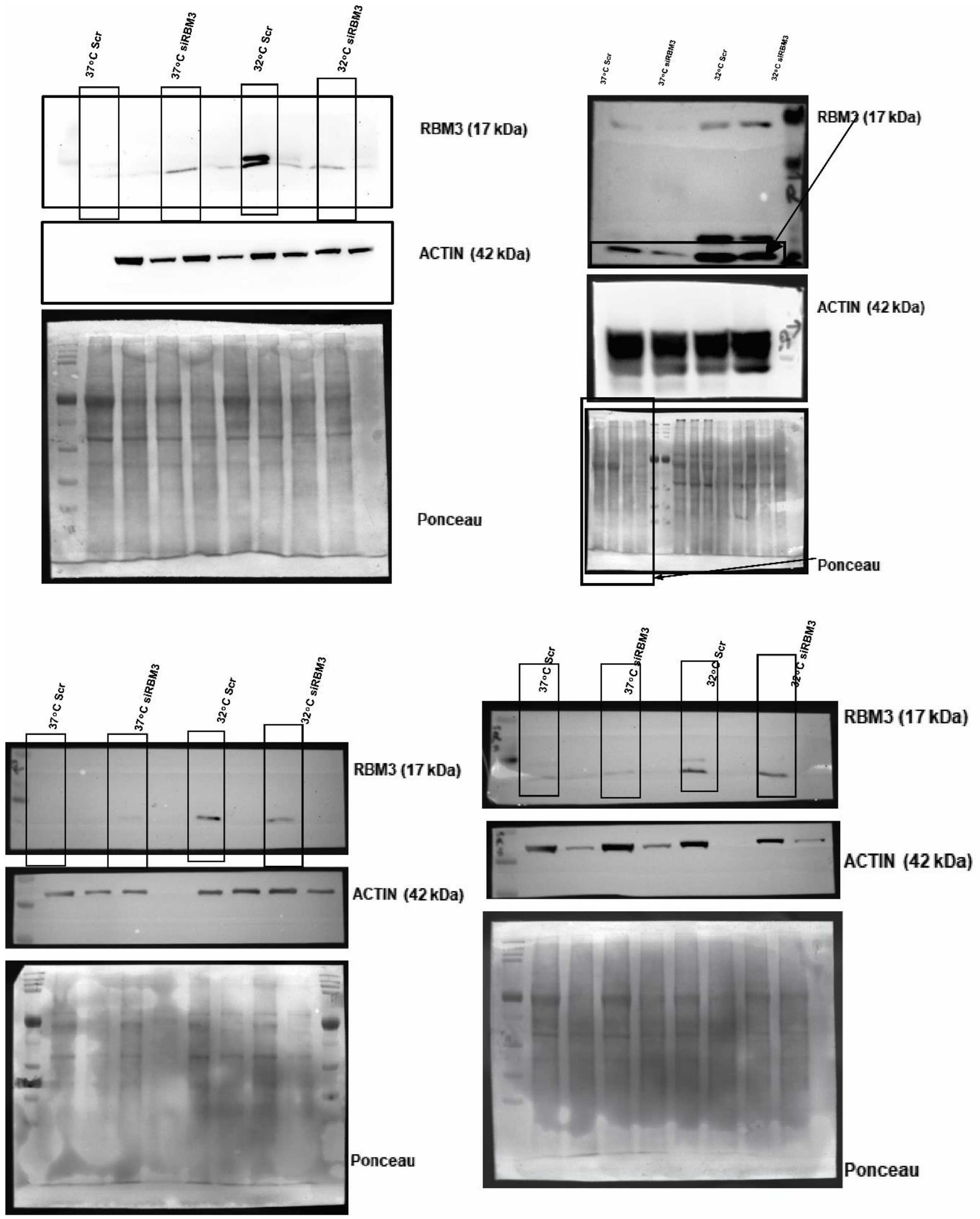

Figure: Uncropped raw western blot images of Fig S3c

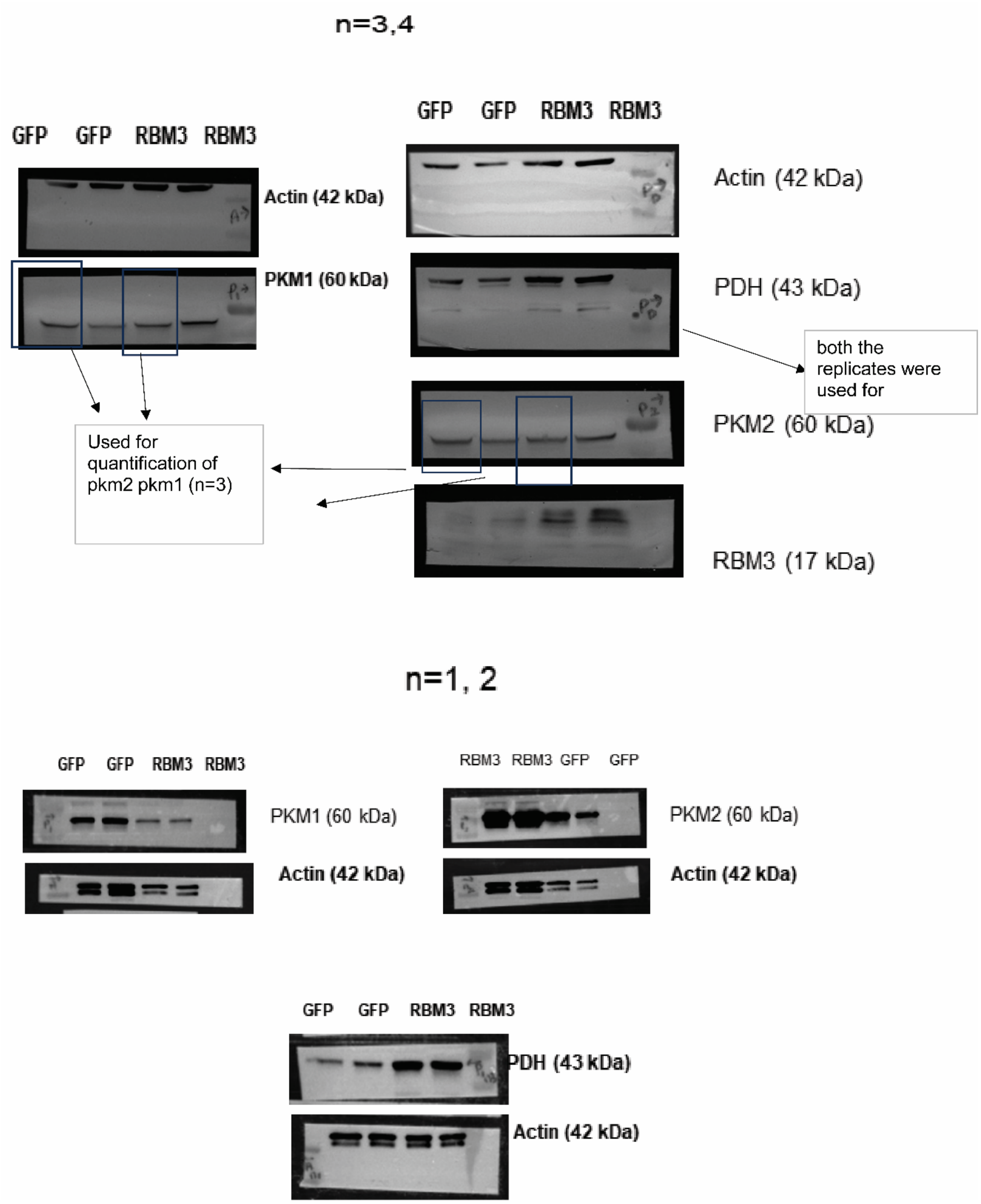

Figure: Uncropped raw western blot images of Fig S4d, S4e

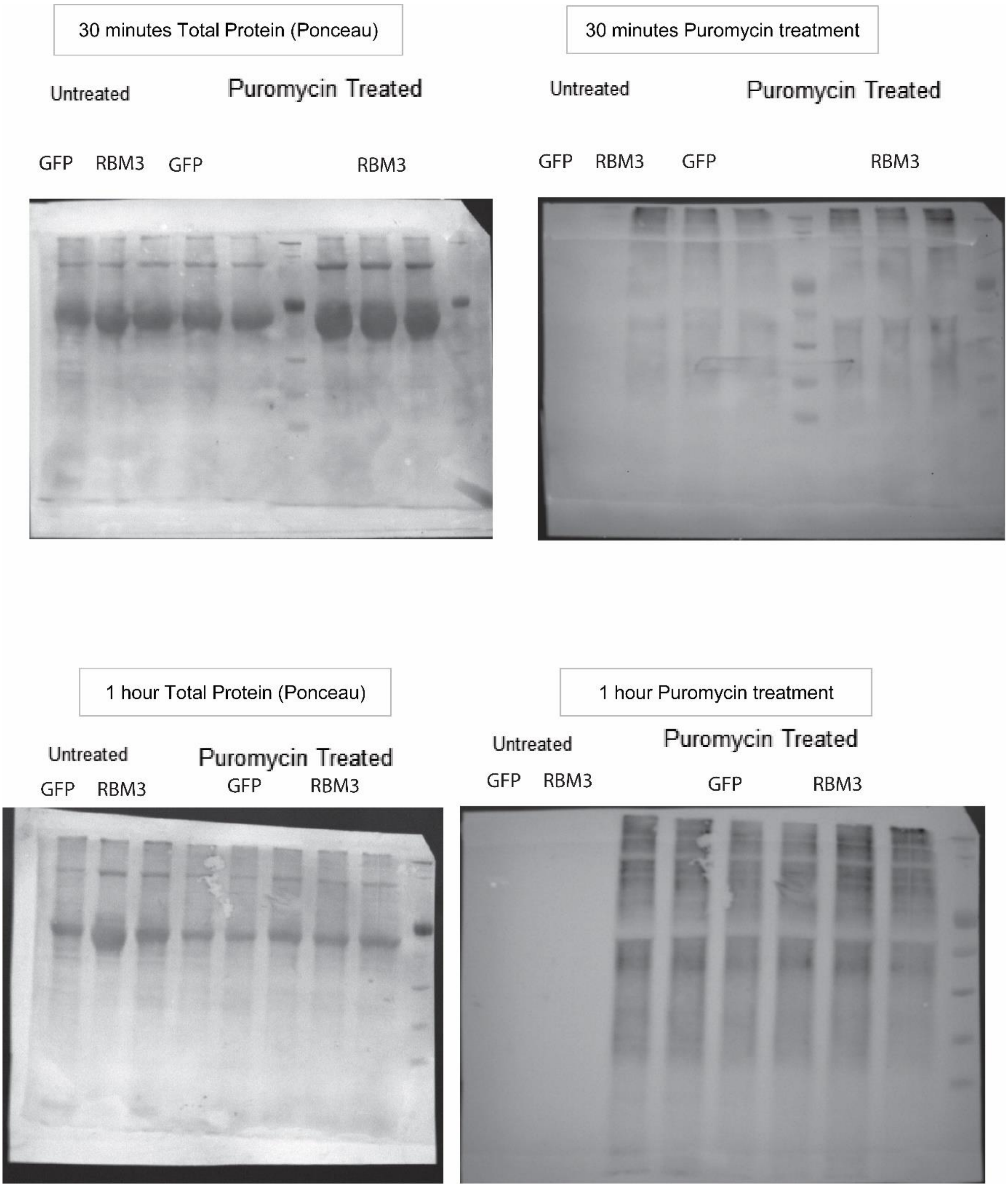

Figure: Uncropped raw western blot images of Fig S7a, S7b, S7c

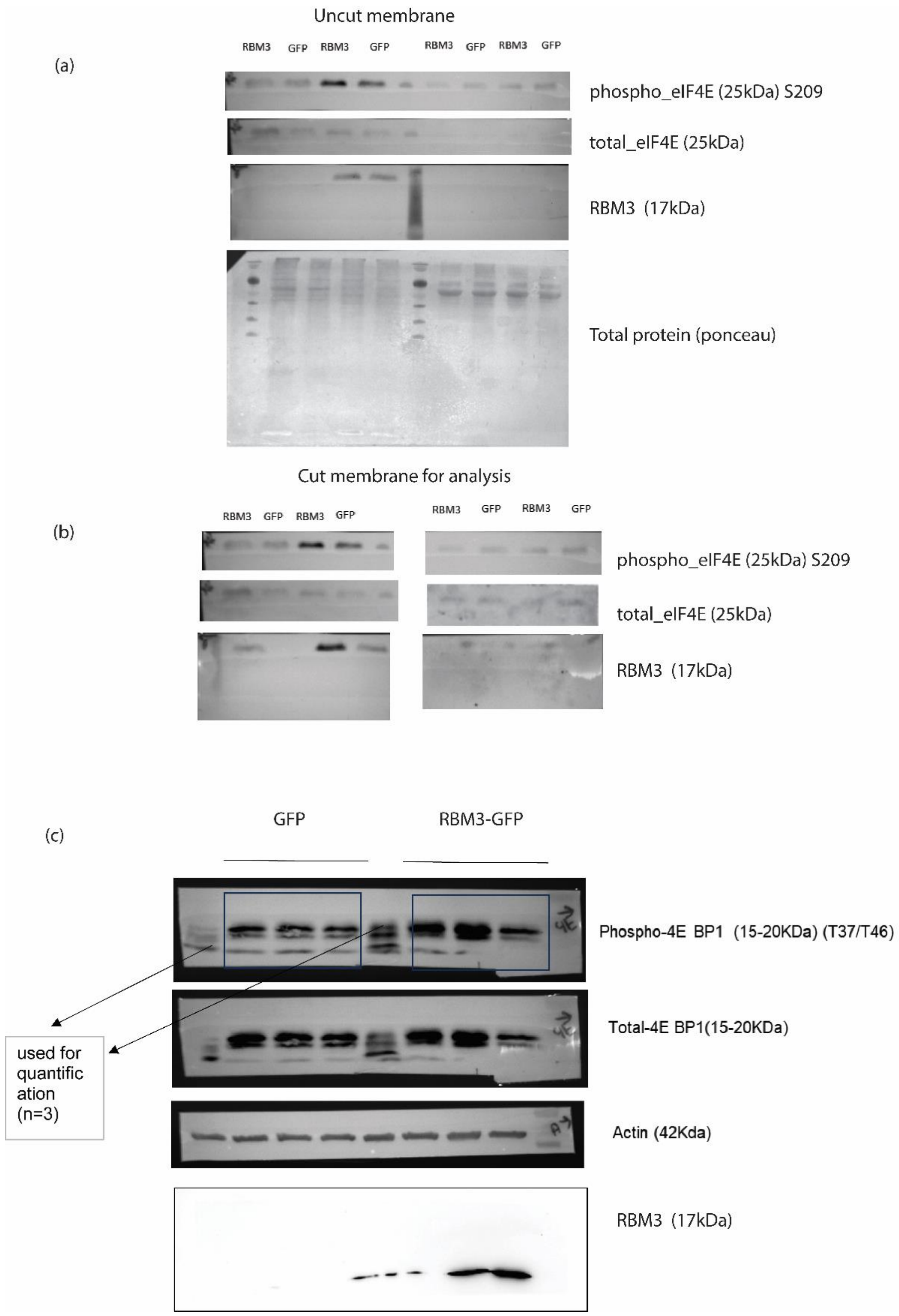

Figure: (a) Uncropped raw western blot images of Fig S7d, S7e (uncut membrane).

(b) Uncropped raw western blot images of Fig S7d, S7e (cut membrane).

(c) Uncropped raw western blot images of Fig S7f, S7g
