# Supplementary material for: Cold-shock proteome of myoblasts reveals role of RBM3 in promotion of mitochondrial metabolism and myoblast differentiation
Source: Commun Biol. 2024 Apr 30;7:515. doi: 10.1038/s42003-024-06196-4 (PMC11061143; doi:10.1038/s42003-024-06196-4)
Supplement: Supplementary file 3 — Description of Additional Supplementary Files [file 42003_2024_6196_MOESM3_ESM.pdf]

Description of Additional Supplementary Files

File name: Supplementary Data 1
Description: Source data behind the graphs and charts in the paper.
